# The evaluation of trace elements of interest in kidney disease in commonly consumed greenhouse vegetables in Isfahan, Iran: preliminary results

**DOI:** 10.12861/jrip.2014.17

**Published:** 2014-04-01

**Authors:** Mohammad Reza Abdi, Khadijeh Rezaee Ebrahim Saraee, Mehdi Rezvani Fard, Jamshid Khorsandi, Milad Baradaran-Ghahfarokhi

**Affiliations:** ^1^Department of Physics, Faculty of Science, University of Isfahan, Isfahan, Iran; ^2^Department of Nuclear Engineering, Faculty of Advance Sciences and Technologies, University of Isfahan, Isfahan, Iran; ^3^Isfahan Miniatori Reactor, Atomic Energy Organization of Iran, Isfahan Nuclear Center, Isfahan, Iran; ^4^Medical Physics and Medical Engineering Department, School of Medicine, Isfahan University of Medical Sciences, Isfahan, 81746-73461, and, Medical Radiation Engineering Department, Faculty of Advanced Sciences & Technologies, Isfahan University, Isfahan, Iran; ^5^Medical Student’s Research Center, School of Medicine, Isfahan University of Medical Sciences, Isfahan, Iran

**Keywords:** Greenhouse vegetables, Kidney disease, Trace elements, Trace minerals

## Abstract

Trace elements play a significant role in biological processes. The aim of this study was to evaluate the trace elements of interest in kidney disease in commonly consumed greenhouse vegetables in Isfahan, Iran. Six kinds of greenhouse vegetables namely; Raphanus sativus (Radish), Cucumis sativus (Cucamber), Solanum lycopersicum (Tomato), green Capsicum annuum (Green bell pepper), yellow Capsicum annuum (Yellow bell pepper) and red Capsicum annuum (Red bell pepper) were collected from Isfahan greenhouses, between December 2012 to March 2013. The vegetables were analyzed in order to determine the concentrations of trace elements and trace minerals using instrumental neutron activation analysis (INAA). The results of INAA showed that, the concentrations of Fe (Iron), Mn (Manganese) and Zn (Zinc) were varied from <10-50.0 mgkg^-1^, 6.8-15.0 mgkg^-1^ and 10.0-23.0 mgkg^-1^, respectively. The elemental concentration of Fe, Mn and Zn in all of the samples were less than the defined tolerable Upper Intake Level (UIL). The results of this study revealed that, considering the measured trace elements and mineral content levels, Isfahan greenhouse vegetables do not impose any serious health harmful effects for individuals in the studied area due to their meal consumptions.

**Implication for health policy/practice/research/medical education:**

Nowadays, greenhouse vegetables and fruits production have attracted considerable attentions, particularly as alternative crops of healthy eating. However, the use of chemical pesticides and fertilizers, as a way of increasing greenhouse vegetables and fruits crops, could endanger the human health and impose harmful effects. In this study, six kinds of greenhouse vegetables namely; Raphanus sativus (Radish), Cucumis sativus (Cucamber), Solanum lycopersicum (Tomato), green Capsicum annuum (Green bell pepper), yellow Capsicum annuum (Yellow bell pepper) and red Capsicum annuum (Red bell pepper) were analyzed. The results of this study revealed that, considering the measured trace elements and mineral content levels, Isfahan greenhouse vegetables do not impose any serious health harmful effects for individuals in the studied area due to their meal consumptions.

## Introduction

Trace elements play an important role in biological processes. They are capable of affecting human health by competing with essential elements for available binding sites and by the activation or inhibition of reactions between metabolic enzymes. Nowadays, greenhouse vegetables and fruits production have attracted considerable attentions, particularly as alternative crops of healthy eating (1). However, the use of chemical pesticides and fertilizers, as a way of increasing greenhouse vegetables and fruits crops, could endanger the human health and impose harmful effects (2).

In Iran, annual meal consumption of fertilizers in agriculture is about 4.5 million tons, and about 87% of these fertilizers is in the form of Phosphorus (3). However, using such amount of fertilizers for more growth in the greenhouses is stated to be carcinogenic (4). In addition to phosphorus, fertilizers contain some other elements such as Arsenic, Chromium, Iron, Manganese and Zinc. It should be noted that, in a definite amounts, these trace elements are needed for the human body and are vital to our health and wellness. However, large amounts of these elements can be toxic for certain individuals. In general, these elements are presented in vegetables and fruits in trace and ultra-trace quantities (2,5).

The aim of this study was to evaluate the trace elements of interest in kidney disease in commonly consumed greenhouse vegetables in Isfahan, Iran. Elements and minerals of interest in this study were Fe (Iron), Mn (Manganese) and Zn (Zinc).

## Materials and Methods

Six types of vegetables namely; Raphanus sativus (Radish), Cucumis sativus (Cucamber), Solanum lycopersicum (Tomato), green Capsicum annuum (Green bell pepper), yellow Capsicum annuum (Yellow bell pepper) and red Capsicum annuum (Red bell pepper) were collected from local Isfahan greenhouses between December 2012 to March 2013. Samples were transported to the laboratory within one day. They were individually brushed to remove adhering soil, washed with tap water, cut into small pieces with a scalpel and transferred to clean, dry vials. After 24 hours freezing, to remove the samples water and to clean and dry they were dried in an oven at 60 ºC for at least 2 days, until a constant dry weight was obtained. The dried samples were homogenized with a pestle and mortar, and then, they were reduced to a powder. For quantity control, four biological standard references of peach leaves NIST-1547, Bovine liver NIST-1577b, rice flour NBS-1568a and apple leaves NIST-1515 were considered (2,6).

Then, the samples were transferred to laboratory of Activation Analysis, Isfahan Research and Fuel Production Center, Isfahan, Iran, for determination of trace elements and minerals concentrations using INAA.

## Results

The results of INAA showed that the concentrations of Fe, Mn and Zn were varied from <10-50.0 mgkg^-1^, 6.8-15.0 mgkg^-1^ and 10.0-23.0 mgkg^-1^, respectively. The elemental concentration of Fe, Mn and Zn in all of the samples were less than the defined tolerable Upper Intake Level (UIL). Table 1  gives the trace minerals concentrations (mgkg-1 dray-weight) in the studied greenhouses vegetables.

**Table 1:** Trace minerals concentrations (mgkg^-1^ dray-weight) in greenhouses vegetables.

Name	Fe (mgkg^-1^)	Mn (mgkg^-1^)	Zn (mgkg^-1^)	Average (mgkg^-1^)
Tomato	<10	6.8±0.3	10.0±1.0	8.4
Cucumber	37.0±9.0	15.0±0.8	23.0±2.0	25
Radish	31.0±8.0	9.7±0.8	13.0±1.0	17.9
Bell pepper (red)	45.0±9.0	11.9±0.4	16.0±1.0	24.3
Bell pepper (green)	50.0±10.0	13.7±0.7	18.0±1.0	27.2
Bell pepper (yellow)	49.0±10.0	10.5±0.5	14.0±1.0	24.5
Min	31	6.8	10	
Max	50	15	23	

## Discussion

In this work, the concentrations of trace elements and trace minerals in vegetable samples from Isfahan greenhouses were investigated. The precision and accuracy of the experiment was tested by analyzing standard reference materials and a good agreement was found. Three elements in samples of greenhouse vegetables were studied.

The values of Fe in the samples were varied between <10 to 50 mgkg^-1^ with the highest value in sample of green bell pepper (50.0 mgkg^-1^) and the lowest in tomato (<10.0 mgkg^-1^). The typical daily dietary intake of iron is 40 mg (7). Iron is an essential mineral element used to transport oxygen to all parts of the body. A slight deficiency in Fe causes anemia and a chronic deficiency can lead to organ failure. Too much Fe leads to production of harmful free radicals, and interferes with metabolism, causing damage to organs like heart and liver (8). The body is able to regulate uptake of Fe, so Fe overdose is rare and usually only occurs when people take supplements. Iron from natural food sources is considered safe and healthy. Previously, the Fe was determined 139, 417, 238, 63 mgkg^-1^, for Iraq pepper, radish, tomato and cucumber, respectively (2). The concentration of Fe in the Malaysian cucumber was 146 mgkg^-1^, reported by Shafaei *et al*. (7).

The values of Mn in the samples varied between 6.8-15.0 mgkg^-1^ with the highest value in sample of cucumber (15.0 mgkg^-1^)and the lowest in tomato (6.8 mgkg^-1^). The Mn content ranging from 20-30 mgkg^-1^ is known as sufficient and toxic or excessive when ranged from 300-500 mgkg^-1^. The typical daily dietary intake of Mn is 30 mg. Manganese is one of the mineral elements which is actively absorbed by plants and has a significant role on the formation of plant mass (9). The concentration of Mn for Malaysian cucumber and southwestern Asia is reported 37.0 and 8.0 mgkg^-1^, respectively (7). Moreover, it is 15.0 mgkg^-1^ for the greenhouse cucumber (7). The Mn was determined 7.0, 3.8, 11.6, and 5.4 mgkg^-1^, for Iraq pepper, radish, tomato and cucumber, respectively (2). The Mn was determined 17.7, 28.2, and 12.9 mgkg^-1^, for Germani pepper, radish and tomato, respectively (9).

Zinc concentrations of the analyzed samples in this study were between 10 mgkg^-1^ (for tomato) and 23 mgkg^-1^ (for cucumber). The Zn was determined 48.7, 74.5, 91.3, and 62.9 mgkg^-1^, for Iraq pepper, radish, tomato and cucumber, respectively (2). The concentration of Zn for Malaysian cucumber is 57.0 mgkg^-1^, while for the greenhouse cucumber it is 23.0 mgkg^-1^ (7). Zinc is an essential mineral required by the body for maintaining a sense of smell, keeping a healthy immune system, building proteins, triggering enzymes, and creating DNA (10). Consuming too much Zn may be disrupted the absorption of copper and iron and also may be created large amounts of toxic free radicals. Conversely, a deficiency in zinc may be lead to the stunted growth, diarrhea, impotence, hair loss, eye and skin lesions, impaired appetite, and depressed immunity (10). The RDA value of zinc element is 40 mg/day. Animal foods are better sources of zinc than plant foods (7). Considering our results, it is clear that the greenhouse vegetables in this research that had zinc concentrations of more than 13 mgkg^-1^ can be considered as high Zn plant foods. The normal range of zinc in healthy vegetables is 20-100 mgkg^-1^ and the toxic or excessive levels of zinc in plants are 100 to 400 mgkg^-1^ (8). Comparing with normal and toxic range, the greenhouses vegetables used in this study were found within sufficiency range rather than the toxic one.

## Conclusion

The concentrations of trace elements and minerals of interest in kidney disease such as: Fe, Mn and Zn in six types of vegetables namely; Raphanus sativus (Radish), Cucumis sativus (Cucamber), Solanum lycopersicum (Tomato), green Capsicum annuum (Green bell pepper), yellow Capsicum annuum (Yellow bell pepper) and red Capsicum annuum (Red bell pepper) grown in Isfahan greenhouses were determined using INAA. The results of this study revealed that, considering the measured trace elements and mineral content levels, Isfahan greenhouse vegetables do not impose any serious health harmful effects on kidney of individuals in the studied area due to their meal consumptions.

## Acknowledgments

The Authors would like to thank the research fellows of the Laboratory of Activation Analysis, Isfahan Research and, Fuel Production Center, for their technical support and advice. We are also deeply grateful to Engineer Shirini who actively took part in all stages of this work.

## Authors’ contributions

All authors wrote the manuscript equally

## Conflict of interests

The authors declared no competing interests.

## Ethical considerations

Ethical issues (including plagiarism, data fabrication, double publication) have been completely observed by the author.

## Funding/Support

None declared.
